# Evaluation of mercury exposure level, clinical diagnosis and treatment for mercury intoxication

**DOI:** 10.1186/s40557-015-0086-8

**Published:** 2016-01-22

**Authors:** Byeong-Jin Ye, Byoung-Gwon Kim, Man-Joong Jeon, Se-Yeong Kim, Hawn-Cheol Kim, Tae-Won Jang, Hong-Jae Chae, Won-Jun Choi, Mi-Na Ha, Young-Seoub Hong

**Affiliations:** Department of Occupational & Environmental Medicine, Dong-A University Hospital, 26, Daesingongwon-ro, Seo-gu, Busan, Republic of Korea; Department of Preventive Medicine, Collage of Medicine, Dong-A University, 32, Daesingongwon-ro, Seo-gu, Busan, Republic of Korea; Heavy-Metal Exposure Environmental Health Center, Dong-A University, 32, Daesingongwon-ro, Seo-gu, Busan, Republic of Korea; Department of Occupational & Environmental Medicine, Collage of Medicine, Young-Nam University, 170, Hyeonchung-ro, Nam-gu, Daegu, Republic of Korea; Department of Occupational & Environmental Medicine & Institute of Environmental and Occupational Medicine, Pusan Paik Hospital, InJe University, 75, Bokji-ro, Busanjin-gu, Busan, Republic of Korea; Department of Occupational & Environmental Medicine, Inha University Hospital, 27, Inhang-Ro, Jung-Gu, Incheon, Republic of Korea; Department of Occupational and Environmental Medicine, College of Medicine, The Catholic University, 222, Banpo-Daero, Seocho-gu, Seoul, Republic of Korea; Department of Occupational and Environmental Medicine, KS Hospital, 220, Wangbeodeul-ro, Gwangsan-gu, Gwangju, Republic of Korea; Department of Occupational & Environmental Medicine, Gachon University Gil Medical Center, 21, Namdong-daero 774beon-gil, Namdong-gu, Incheon, Republic of Korea; Department of Preventive Medicine, College of Medicine, Dankook University, 119, Dandae-ro, Dongnam-gu, Cheonan-si, Chungcheongnam-do, Republic of Korea

**Keywords:** Mercury, Intoxication, Diagnosis, Treatment

## Abstract

Mercury occurs in various chemical forms, and it is different to health effects according to chemical forms. In consideration of the point, the evaluation of the mercury exposure to human distinguished from occupational and environmental exposure.

With strict to manage occupational exposure in factory, it is declined mercury intoxication cases by metallic and inorganic mercury inhalation to occupational exposure. It is increasing to importance in environmental exposure and public health.

The focus on the health impact of exposure to mercury is more on chronic, low or moderate grade exposure—albeit a topic of great controversy—, not high concentration exposure by methylmercury, which caused Minamata disease.

Recently, the issue of mercury toxicity according to the mercury exposure level, health effects as well as the determination of what mercury levels affect health are in the spotlight and under active discussion. Evaluating the health effects and Biomarker of mercury exposure and establishing diagnosis and treatment standards are very difficult.

It can implement that evaluating mercury exposure level for diagnosis by a provocation test uses chelating agent and conducting to appropriate therapy according to the result. but, indications for the therapy of chelating agents with mercury exposure have not yet been fully established.

The therapy to symptomatic patients with mercury poisoning is chelating agents, combination therapy with chelating agents, plasma exchange, hemodialysis, plasmapheresis. But the further evaluations are necessary for the effects and side effects with each therapy.

## Background

Mercury has been used for nearly 3000 years of human history. The metal is still widely used in many areas, including making thermometers, barometers, manometers, whitening cosmetics, and dental amalgam. Occupational mercury intoxication occurred frequently in the workers of the hat manufacturing factories of Europe in the nineteenth century. In Korea, victims of mercury poisoning due to occupational exposure to a high concentration were seen in the thermometer manufacturing, fluorescent lamp manufacturing, and waste reprocessing industries from the 1980s to the early 2000s. Recently, stricter environmental management of worksites reduced mercury intoxication cases caused by inhalation of metal and inorganic mercury in workplaces. However, given that many workers are still being exposed to mercury in various occupations, the possibility of mercury intoxication by occupational exposure still exists, and interventions are necessary for such cases.

The health hazards of mercury became well known and gained worldwide attention due to the 1956 outbreak of Minamata disease in Japan. In the incident, methylmercury, formed as a byproduct of chemical compound (acetaldehyde) production in a fertilizer factory, was released to the nearby shore for a long time, which polluted the marine ecosystem including the fish and shellfish. About 5000 of the residents who ate seafood from the area died or suffered injury. Later, damage cases caused by the mercury were also reported in Iraq, Iran, and Tanzania. Recently, the significance of mercury as an environmental toxin has been highlighted, as seen in the conclusion of the Minamata Convention on Mercury. International cooperation has also gained increased importance for addressing the problem of mercury, since the metal migrates and circulates throughout the globe.

The global mercury issue is drawing more attention in Korea, given that the level of human exposure to mercury in the country has been rated high. According to the 2009 and 2010 Korea National Health and Nutrition Examination Survey (KNHANES), the geometric means of the blood mercury concentration levels in Korean adults were 4.28 ug/L and 3.64 ug/L in 2009 and 2010, respectively. Furthermore, the 2009 and 2010 Korean National Environmental Health Survey reported the geometric means of the blood mercury concentration levels to be 3.93 ug/L and 2.88 ug/L, respectively. Despite varying levels reported among the surveys, blood mercury concentration levels in Korea are reported to range from 2.88 ug/L to 4.28 ug/L. That is about four to six times higher than that of the U.S. (0.86 ug/L), Germany(0.58 μg/L) or Canada (0.76 ug/L) [1–3]. Furthermore, considering that about 26.2 % of the population in Korea is estimated to exceed the limit of the blood mercury exposure level set by the U.S. Environmental Protection Agency (EPA) [4], Korea shows a much higher exposure level to mercury than do advanced countries.

The recent focus on the health impact of exposure to mercury is more on chronic, low or moderate grade exposure—albeit a topic of great controversy—, not high concentration exposure by methylmercury, which caused Minamata disease, as in the past. The results of the three key epidemiological studies conducted in the Faroe Islands, Seychelles, and New Zealand, which aimed at evaluating the health impact of mercury, have great implications for the danger of low grade mercury exposure. Nevertheless, the health hazards of mercury manifest themselves in widely varying forms. In addition, treatment of mercury intoxication caused by mercury exposure has received little research attention. Recently, the issue of mercury toxicity according to the mercury exposure level as well as the determination of what mercury levels affect health are in the spotlight and under active discussion. In this study, we review the recent research findings on the methods of diagnosis and treatment of mercury poisoning according to the exposure level of mercury.

## Diagnosis for mercury intoxication

### General characteristics of mercury

Mercury occurs in several forms including metallic, inorganic, and organic compounds. Its patterns in terms of ecosystem circulation, accumulation, exposure to the human body, biological effects, and toxicity depend on the characteristics of the different mercury forms. Metallic mercury is commonly absorbed through the respiratory tract. Because it forms globules, it is hardly absorbed in the gastrointestinal (GI) tract, and thus is harmless when taken orally. In addition, being fat soluble, it readily passes through the alveolar cell barrier and oxidizes to inorganic mercury, and combines with protein and exerts a cumulative effect. The mercury absorbed in the body mainly goes to the kidneys and brain. The half-life of mercury in the body is about 70 days.

Inorganic mercury is mainly absorbed through the respiratory tract, yet to a small extent is also absorbed through the skin (3–4 %) or GI tract (2–10 %). However, unlike metallic mercury, inorganic mercury cannot penetrate the blood–brain barrier and commonly ends up building up in the kidney, the target organ. The main excretory pathways include the urine and feces, with about two months’ half-life. Inorganic mercury discharged to the natural environment flows into the sea, river, or stream. In the water, it converts into metallic mercury by bacteria and plankton. Then it accumulates in the body of aquatic organisms including fish and shellfish. Finally, when humans eat the seafood, the mercury enters the human body in the form of metallic mercury. Methylmercury, a major type of organic mercury, exerts fatal toxic effects on the human body. In the natural environment, it is commonly found in the form of monomethylmercury and dimethylmercury. Methylmercury is easily absorbed into the GI tract (≥95 %) and into the respiratory tract (≈80 %). About 90 % of methylmercury is excreted to the feces via bile, and less than 10 % to the urine. The absorbed mercury is distributed throughout all tissues within 30 h. Its half-life ranges from 45 to 70 days. As both metallic and organic mercury easily cross the blood–brain barrier and placenta, they can be excreted in breast milk and transmitted to the fetus.

### Biological pathway to exposure of mercury

The pathways of exposure to mercury include the following: (a) ingestion of food, (b) inhalation or absorption (via the skin) of mercury vapor at worksites, (c) exposure during the processing of industrial and household wastes, and (d) use of pharmaceutical drugs or cosmetics. Similarly, mercury which passes into the human body through pathways such as the digestive system, respiratory system, and the skin, is absorbed within the body in varying rates, depending on the form of the mercury compound. In 2012, the Korea Food and Drug Administration (KFDA) reported on the exposure contribution rate by medium in different age groups, based on the data on mercury concentrations in food. According to the report, the exposure contribution rate of inhalation from the air was 0.47–0.83 %, ingestion of drinking water (tap water) was 0.01–0.02 %, and ingestion of soil, 0.03–0.32 %; all of them were highest in infants and toddlers. The study also reported that the major medium of mercury exposure was food, as shown by the fact that mercury exposure by ingestion of food accounted for 98.85–99.48 % [5].

### Biological indices of mercury exposure

“Biomarker” is a widely used term, signifying an indicator of a condition of the body or biological samples. Biomarkers are largely categorized into markers of exposure, markers of effect, and markers of susceptibility (NAS/NRC, 1989). The mercury absorbed in the body builds up in each type of tissue, including the hair, distributed through the blood. Therefore, the most accurate way to measure the amount of mercury in the body would be measuring mercury concentration levels in each organ tissue. However, as this would involve great difficulty, the common method of mercury measurement is to use samples of the blood, urine, hair, or fingernail. According to studies on occupational mercury exposure, recent exposure to mercury is reflected in the blood and urine mercury concentration levels [6, 7]. Nonetheless, in remarkably low concentration levels of mercury exposure, the association between the exposure and blood or urine mercury concentration levels was low [8]. The mean mercury concentration levels in the general population in whole blood and urine were observed to be 1–8 μg /L and 4–5 μg /L, respectively [7]. Recently, the International Commission on Occupational Health (ICOH) and the International Union of Pure and Applied Chemistry (IUPAC) set the average blood mercury concentration level in those who do not eat fish at 2 μg /L. Such blood and urine mercury concentration levels signify background concentrations, the mean concentration level in the general population.

#### Blood mercury concentration

Blood mercury concentrations rapidly increase immediately after or during brief exposure. Therefore, the measurement also needs to take place right after the exposure [9]. However, in cases of those who have been chronically exposed to mercury, blood mercury concentration levels maintained a high level even when the exposure has ceased, due to the heavy burden of mercury on the body [8].

In the meantime, the concentration level of methylmercury—which accounts for the largest share in the blood—is known to be linked with the amount of mercury we are exposed to daily, according to the equation in Fig. 1 [7]. Furthermore, although the concentration level of methylmercury in red blood cells is high in acute poisoning, it varies widely in chronic intoxication. The mercury concentration in whole blood is usually lower than 10 μg/L, but the value of 20 μg/L or below is considered normal. The blood mercury concentration can rise to 35 μg/L after long-term exposure to mercury vapor [10].

#### Urine mercury concentration

Urine mercury concentration is very stable and relatively simple, due to the characteristic of the medium. Moreover, it is a quick means of identifying those exposed to mercury. However, because organic mercury represents a very small portion of urine mercury, urine mercury is more useful for the analysis of metallic or inorganic mercury compounds. In addition, workers exposed to mercury over the long term exhibit high levels of urine mercury concentration for a long period of time, as seen in the blood mercury concentration, due to a burden of mercury on the body [10]. When the urine mercury concentration exceeds 100 μg/L, neurological symptoms can develop, and the level of 800 μg/L or above can be fatal. Organic mercury such as methylmercury is usually excreted to the feces. Hence, urine concentration levels cannot reflect organic mercury concentration levels of the body [11].

#### Hair mercury concentration

While 80–90 % of hair is composed of keratin, which contains an amino acid group high in sulfhydryl groups and thus easily combines with metal. Therefore, with exposure to mercury, the mercury concentration can become high in hair, and hair can show the level of mercury exposure that has occurred over a long period of time. Hair mercury concentration is also known to be proportionate to blood mercury concentration. Such an association can be applied to evaluate blood mercury concentration by measuring hair mercury concentration. The migration of mercury to hair is irreversible. Therefore, a decrease in hair mercury concentration only occurs when hair falls out of the scalp {Nielsen, 1991 #721}. Hair mercury concentration is used as a biomarker of chronic exposure to methylmercury. Once mercury combines with hair, it never separates and remains consistently [12–14]. Extensive studies have been performed on the association between hair mercury concentration and blood mercury concentration [15–21]. After exposure to methylmercury, total mercury levels in hair and blood can be used as biomarkers of mercury intoxication. The hair-to-blood mercury concentration ratios set by the U.S. Food and Drug Administration (FDA) and the World Health Organization (WHO) are 250:1 [22] and 250–300: 1 [23], respectively. Similarly, the ratio set by a 2010 Korean study on 1200 residents of a region was about 278.5:1 [24]. The internationally recommended limit of hair mercury concentration is 1 mg/kg as proposed by the WHO. However, hair mercury concentration analysis results alone cannot be used to discern whether one is exposed to or intoxicated by mercury. Generally, hair mercury concentration does not exceed 10 mg/kg. However, in moderate mercury poisoning, the concentration level ranges between 200 and 800 mg/kg, and in severe intoxication it goes up to 2400 mg/kg. The WHO recommends the monitoring of methylmercury concentration in pregnant women’s hair and argues that the level of 10 ppm or above can increase the risk of fetal neurological defects [25].

### Other clinical laboratory tests

Mercury occurs in various physical and chemical forms and can convert from one form to another inside the body. In general outpatient settings, blood and urine mercury concentrations generally indicate the total mercury concentration level, without indicating the concentrations of organic, non-organic, or metallic mercury specifically [26].

The general laboratory tests to evaluate mercury intoxication include complete blood cell count, electrolyte assays, and renal and hepatic function tests. Electrocardiography (ECG), pulmonary function test (PFT), cardiovascular monitoring, electroneuromyography, and neuropsychological tests are also used for the evaluation [10]. Regarding the lab tests to evaluate the influence of mercury exposure to health, the results of the tests which examine abnormalities in the neurological or kidney function that show mercury toxicity have an association with blood or urine mercury concentration. Nonetheless, most lab tests did not exhibit mercury specificity and thus the results were considered to be affected by other factors. There have been many studies on the association between kidney function or neurological changes and urine mercury concentration. Malfunction in the kidney was widely studied as a sensitive indicator of mercury exposure. However, in numerous studies, renal function parameters were interdependent [27]. The neurophysiological or neuropsychiatric influence of mercury has been extensively researched on the individuals occupationally exposed to mercury. The neurological changes induced by mercury have been demonstrated to be similar to those of other chemical elements affecting the brain. The association between urine mercury concentration and performance in a memory test and language intelligence test has been established [28]. The possible biomarkers of autoimmune effects of mercury include anti-glomerular basement membrane antibody, anti-DNA antibody, serum immunoglobulin E compounds, and total immunoglobulin E [29].

### Guidelines for mercury exposure level

#### Workplace environment

Mercury exposure at workplaces handling mercury is the most critical and dangerous exposure channel. The mercury exposure limit at workplaces is set by each country, according to its occupational exposure limits. In Korea, the Ministry of Employment and Labor established the exposure limit for the workplace environment in 2013 as the following: time weighted average (TWA) for aryl mercury compounds: 0.1 mg/m^3^; TWA for alkyl mercury compounds: 0.01 mg/m^3^; short term exposure limit (STEL): 0.03 mg/m^3^; and TWA for other substances, excluding aryl and alkyl compounds: 0.025 mg/m^3^ [30].

#### General environment

The mercury exposure limit in the general environment also varies. Mercury should not be found in water, and the limit of mercury compounds is set to be 5 mg/m^3^ or lower in the air. The worrisome level (WL) of soil contamination in farmland and industrial areas or factories is suggested to be 4 mg/kg and 16 mg/kg, respectively. The soil contamination countermeasure standard (CS) in farmlands and industrial areas or factories is 10 mg/kg and 40 mg/kg, respectively. Regarding mercury in food, Europe, international bodies, and developed countries suggest varying permissible limits. The EU suggests a maximum level (ML) for mercury, while the U.S. suggests limits on mercury as a harmful substance. However, in general, the permissible limits are suggested for seafood and limited to only some processed foods. For permissible limits of mercury exposure to the human body, international organizations and advanced countries set provisional tolerable weekly intake (PTWI) for mercury. They tend to reinforce the limits for methylmercury. The U.S. and Netherlands propose the PTWI to be 0.7 μg/kg body weight/week, the lowest among different countries, while Japan suggests a higher methylmercury exposure limit than others, which is 2.0 μg/kg body weight/week.

### Diagnosis criteria for mercury intoxication

Evaluating the influence of mercury exposure to health and establishing treatment standards are very difficult. In general, the health effects of mercury exposure have been studied and reported with the focus on occupational exposure and exposure due to accidents. Studies on the mercury exposure levels in the general population have been conducted using various methodologies, and in many cases, exposure to mercury via food ingestion implies the greatest possibility of health consequences among exposure pathways. Health impacts from food occur with very low concentrations of mercury and usually do not warrant treatment. Thus, the mercury levels requiring treatment in cases of mercury exposure by food ingestion should be set higher than the threshold mercury concentration level known to affect health, and the treatment strategy should be implemented accordingly.

Different studies have recommended varying mercury concentration thresholds at which health impacts of mercury exposure should be evaluated. A study that reviewed several sets of data including those of the 2005 U.S. Centers for Disease Control and Prevention (CDC) [31], 2004 U.S. EPA [32], and 2001 American Conference of Governmental Industrial Hygienists (ACGIH) [33] established the normal mercury concentration range to be 4.6 μg/L and 4.0 nmol Hg per mmol creatinine and below, for blood mercury and urine mercury, respectively, which are the 95th percentile in normal adults.

The study also suggested the mercury limit for vigilance regarding meaningful exposure sources such as occupational exposure or food ingestion and for setting reduction measures to be 10.0 μg/L and 19.8 nmol Hg per mmol creatinine, for blood mercury and urine mercury, respectively. The concentration level which warrants clinical interventions was proposed to be 40.0 μg/L or higher for the blood mercury level. For the urine mercury concentration alone, the data was insufficient; thus the limit for the concentration level to initiate treatment was not suggested [34]. Another study also noted that interventions need to be considered for cases in which mercury-related symptoms exist and the blood mercury concentration is 100 μg/L or higher, as well as asymptomatic cases with 200 μg/L or higher blood mercury concentrations [35].

To date, there are no universal diagnostic criteria for mercury overload. A review in the U.S. in 2012 suggested that when mercury is excreted in the urine at levels in the NHANES reference range of 2 standard deviations or higher by a provocation test, the victim is overexposed to mercury [36]. The biological exposure parameter for mercury, as set by the U.S. federal Biological Exposure Index (BEI) is 50 microgram/L in the urine. This reflects the present or recent exposure level, not the mercury body burden. Some studies have reported meaningful toxicity symptoms at 50 microgram/L or lower concentrations [37, 38]. According to Kazantzis, it has been impossible to establish the mercury concentration level in blood or urine that manifests mercury-related physical symptoms [39]. While establishing mercury concentration levels in blood or urine that warrant treatment is difficult, a provocation test is considered to be a reliable tool. The test uses chelating agents such as 2,3 dimercapto-1-propanesulfonate (DMPS) and can reflect the accumulated amount of mercury in the body [40]. Indications for a provocation test are as follows: (1) Does the victim exhibit various and unclear symptoms related to mercury exposure? (2) Has the victim overeaten seafood, had amalgam fillings, or been vaccinated with substances containing thimerosal? (3) Has the victim been occupationally exposed to mercury? (4) Does the victim have a family history of Alzheimer’s disease, Parkinson’s disease, or diseases related to mercury exposure? (5) Does the victim have glutathione transferase (GST) polymorphisms?

## Treatment for mercury intoxication

### General supportive treatment

When mercury poisoning is suspected, an interview with a victim to take a detailed history of environmental exposure to mercury, an occupational history, and physical examination all need to be undertaken. Whether the victim had chronic or acute exposure needs to be determined, and identification of a mercury exposure source and removal of the source must be carried out. The victim’s airway, respiratory and cardiovascular status should also be evaluated. If necessary, clothing should be removed. In case of skin exposure, the skin needs to be washed with water and soap. If the eyes were exposed, cleansing with saline is necessary [41]. In case a person has drunk or inhaled mercury, the respiratory status of the victim including oxygen saturation needs to be carefully observed. If the victim exhibits symptoms such as difficulty in breathing, a chest X-ray and arterial blood gas analysis (ABGA) need to be performed, and intubation and artificial ventilation should be prepared [41]. If intubation is to be performed, eliminating liquid mercury through tracheal suction is helpful in removing contamination.

In case inorganic mercury compounds (H_0_) were ingested and there is no vomiting or aspiration, observation and symptom management are needed until mercury is excreted out of the body. At the same time, a kidney-ureter-bladder X-ray (KUB) may be used to track movement of mercury through the GI tract. In case of ingestion of inorganic mercury compounds such as mercury (II) chloride (HgCl_2_), the condition needs to be treated the same way as in a case of corrosive substance ingestion, due to the risk of damaging and perforating the GI tract mucosa. In case of acute GI poisoning, symptomatic treatment such as evaluation of the airway, respiration, and cardiovascular status is essential. Extensive intravenous therapy is necessary for a symptomatic victim. Collaboration among the ear, nose, throat (ENT), general surgery, and gastroenterology departments is also needed. In addition, the condition of the laryngopharynx and GI tract needs to be checked in a fasting state. If the examined areas are intact, gastric irrigation using charcoal should be performed [42]. Finally, blood dialysis is an essential intervention in treating kidney failure caused by inorganic mercury intoxication. For blood dialysis, evaluation of kidney functioning should be performed.

### Treatment with chelating agents

Symptomatic patients with mercury poisoning warrant immediate treatment with chelating agents. However, it is unclear whether therapy with chelating agents is truly beneficial in severely intoxicated patients. Furthermore, indications for the therapy have not yet been fully established.

Chelating agents that can be used for acute inorganic mercury (Hg0 or Hg++) poisoning include dimercaprol (British Anti-Lewisite, BAL), D-penicillamine (DPCN), dimercaptopropane sulfonate (DMPS), and succimer (dimercaptosuccinic acid, DMSA). Currently, no chelation agents have been approved for methylmercury or ethylmercury by the U.S. FDA**.** Notably, BAL is absolutely contraindicated for the treatment of organic mercury intoxication [43].

DPCN is a water-soluble derivative of penicillin. It increases the excretion of lead and mercury through the urine. For mercury poisoning, adults are treated with 250 mg of DPCN orally, four times a day over one to two weeks. Children are treated with 20–30 mg/kg of DPCN (maximum 250 mg/dose) daily divided into four doses. However, DPCN is used only for metallic and inorganic mercury poisoning, not for organic mercury poisoning [44]. Adverse effects of the agent include leukopenia, thrombopenia, aplastic anemia, proteinuria, hematuresis, and nephrotic syndrome. Recently, DMSA has frequently been used to replace DPCN, given its strong ability to transport metal and fewer side effects. The agent needs to be used with care [10].

Dimercaprol, or BAL, can be administered by intramuscular injection only, which causes pain. The agent has a narrow therapeutic window and a risk of allergic reaction. Recent substitutes for BAL include DMSA and DMPS. It is highly water-soluble and can be administered orally, intravenously, rectally, and percutaneously.

DMSA has a half-life of about three hours, which can be prolonged in children and those who have mercury poisoning. When administered orally, its absorption rate is about 20 %. The agent increases urine excretion of methylmercury and inorganic mercury. In animal tests, it was found to be effective in promoting the excretion of methylmercury from the brain. Accordingly, it is the most commonly used chelating agent for severe methylmercury poisoning. It can be taken orally and its adverse effects can be minimized. While mucocutaneous eruption and toxic epidermal necrosis may occur very rarely as adverse effects, the conditions resolve when administration of the drug is stopped [45].

The absorption rate of DMPS is about 39 % when taken orally, which is higher than that of DMSA. As DMPS is also more stable than DMSA, it is more frequently given intravenously. Its half-life is around 20 h after intravenous administration. DMPS also promotes the excretion of methylmercury and inorganic mercury in the urine. In animal tests, DMPS was more effective in removing methylmercury through the kidneys than DMSA. However, DMPS was not effective in removing methylmercury from the brain [46]. The following is an overview of the chelating drugs for mercury intoxication (Table 1).

**Table 1:** Overview of chelation drugs for mercury intoxication

Chemical name (common names, abbreviations)	Dose	Adverse effect	Elements chelated
2,3-bis(sulfanyl)butanedioic acid (Dimercaptosuccinic acid; DMSA; Succimer)	10 mg/kg (or 350 mg/m^2^) q 8 h for 5 days, then 10 mg/kg q 12 h for 14 days (a total of 19 days), P.O.	Gastrointestinal disturbances, mild increase in serum transaminase	Lead, arsenic, mercury, cadmium, silver, tin, copper
Sodium 2,3-bis(sulfanyl)propane-1-sulfonate (Sodium (Dimercaptopropanesulfonate; DMPS; Dimaval)	5 mg/kg q 6–8 h, P.O., I.M.,I.V., SQ	Low back (kidney) pain, gastrointestinal disturbances, skin rash, fatigue, hypersensitivity reactions	Mercury, arsenic, lead, cadmium, tin, silver, copper selenium, zinc, magnesium
	Children: 5-day courses of 200 or 400 mg of DMPS per m^2^ surface area per day		
(2S)-2-amino-3-methyl-3-sulfanylbutanoic acid (3-Sulfanyl-D-valine; Penicillamine; D- Penicillamine)	10 mg/kg/d for 7 days with a possibility of a prolonged treatment during 2 to 3 weeks, P.O.	Interstitial nephritis, hypersensitivity reactions, gastrointestinal disturbances, leukopenia and thrombocytopenia	Copper, arsenic, zinc, mercury, lead
2,3-bis(sulfanyl)propan-1-ol (Dimercaprol; British Anti-Lewisite; BAL)	50–75 mg/m^2^ q 4 h for 5 days, deep I.M.	Allergy, gastrointestinal symptoms, tachycardia, fever, elevation of liver function tests	Arsenic, gold, mercury, lead (BAL in combination with)

### Combination therapy with chelating agents

One of the important topics related to heavy metal toxicity treatment at present is combination therapy. Administering DMSA and MiADMSA concurrently was found to be more effective than administration of MiADMSA alone. Not only that, the combination therapy was effective in controlling lipid peroxidation as well as the activity level of catalase. Such effects also help reduce the amount of chelating agents, promote better clinical recovery, and minimize side effects [47].

### Plasma exchange-hemodialysis-plasmapheresis

Plasma exchange should be initiated within 24–36 h from the diagnosis of mercury poisoning, when a patient’s life is at risk and there is no other stable treatment alternative. Hemodialysis is the best option when a pollutant is hydrosoluble and dialyzable. It is also necessary when a victim develops renal failure. Nonetheless, some toxic substances bind with plasma proteins and are difficult to separate through hemodialysis. Plasmapheresis removes heavy metals bound with protein in plasma, such as mercury [48]. In treatments involving chelating agents alone, the elimination half-life of inorganic mercury ranges from about 30 to 100 days. In comparison, when DMPS and hemodialysis are used together, the elimination half-life decreases to two to eight days [49].

## Conclusions

Mercury is an environmental toxin commonplace in the general environment with increasing importance in recent years. It has been determined that it is not only harmful to the health of vulnerable populations such as pregnant women and children, but is also toxic to ordinary adults in various ways. Recent studies consistently suggest that chronic exposure even to low concentration levels of mercury can cause cardiovascular toxicity, reproductive and developmental toxicity, neurotoxicity, nephrotoxicity, immunotoxicity, and carcinogenicity. In this study, considering all these characteristics of mercury, we evaluated the proposed mercury exposure thresholds based on recent research findings, and reviewed both the current national and international understanding of the diagnosis and treatment of mercury intoxication. We hope this study will be helpful for management, diagnosis, and treatment of mercury exposure.
